# Identification of Long Non-coding RNA Isolated From Naturally Infected Macrophages and Associated With Bovine Johne's Disease in Canadian Holstein Using a Combination of Neural Networks and Logistic Regression

**DOI:** 10.3389/fvets.2021.639053

**Published:** 2021-04-22

**Authors:** Andrew Marete, Olivier Ariel, Eveline Ibeagha-Awemu, Nathalie Bissonnette

**Affiliations:** ^1^Agriculture and Agri-Food Canada, Sherbrooke Research and Development Centre, Sherbrooke, QC, Canada; ^2^Faculty of Science, Sherbrooke University, Sherbrooke, QC, Canada

**Keywords:** bovine, genomics, long non-coding RNA, macrophages, paratuberculosis (*Mycobacterium avium* ssp. *paratuberculosis*), Johne's disease, MAP disease

## Abstract

*Mycobacterium avium* ssp. *paratuberculosis* (MAP) causes chronic enteritis in most ruminants. The pathogen MAP causes Johne's disease (JD), a chronic, incurable, wasting disease. Weight loss, diarrhea, and a gradual drop in milk production characterize the disease's clinical phase, culminating in death. Several studies have characterized long non-coding RNA (lncRNA) in bovine tissues, and a previous study characterizes (lncRNA) in macrophages infected with MAP *in vitro*. In this study, we aim to characterize the lncRNA in macrophages from cows naturally infected with MAP. From 15 herds, feces and blood samples were collected for each cow older than 24 months, twice yearly over 3–5 years. Paired samples were analyzed by fecal PCR and blood ELISA. We used RNA-seq data to study lncRNA in macrophages from 33 JD(+) and 33 JD(–) dairy cows. We performed RNA-seq analysis using the “new Tuxedo” suite. We characterized lncRNA using logistic regression and multilayered neural networks and used DESeq2 for differential expression analysis and Panther and Reactome classification systems for gene ontology (GO) analysis. The study identified 13,301 lncRNA, 605 of which were novel lncRNA. We found seven genes close to differentially expressed lncRNA, including *CCDC174, ERI1, FZD1, TWSG1, ZBTB38, ZNF814*, and *ZSCAN4*. None of the genes associated with susceptibility to JD have been cited in the literature. LncRNA target genes were significantly enriched for biological process GO terms involved in immunity and nucleic acid regulation. These include the MyD88 pathway (*TLR5*), GO:0043312 (neutrophil degranulation), GO:0002446 (neutrophil-mediated immunity), and GO:0042119 (neutrophil activation). These results identified lncRNA with potential roles in host immunity and potential candidate genes and pathways through which lncRNA might function in response to MAP infection.

## Introduction

One of the most economically significant diseases in livestock is paratuberculosis (1). The etiological agent of paratuberculosis or Johne's disease (JD) is *Mycobacterium avium* ssp. *paratuberculosis* (MAP). Whitlock and Buergelt described JD as chronic, wasting, incurable, and infectious ruminant enteritis (2). For dairy producers, MAP infection translates to significant financial losses related to reduced milk production, decreased pregnancy rates, increased replacement costs, and decreased slaughtered carcass weight (3, 4), not to mention diminished animal welfare. Around 24–66% of dairy herds in Canada are MAP infected (5). Horizontal transmission of infection via the fecal–oral route is the most important mode of spread of infection due to the high amounts of MAP excreted in the feces. After ingestion of contaminated water or food, MAP reaches the gastrointestinal tract; MAP shows an evident tropism for this site (1, 6). The first route of MAP entry is through the ileum and jejunum's organized lymphoid tissue, the Peyer's patch in the intestinal mucosa and submucosa (7). These early events of MAP infection occur in two functional stages: (1) invasion through the intestinal barrier via MAP discharge from epithelial M cells and (2) phagocytosis and survival in macrophages of the lamina propria (7–9). It is known that MAP uses tissue-resident macrophages as its primary reservoir for survival and multiplication (10–12). Interestingly, genetic variations in numerous candidate genes expressed in macrophages are associated with resistance/susceptibility to MAP infection, notably the *NOD2* (13, 14), *IL10* (15–18), *SLC11A1*, and Toll-like receptor genes (19, 20).

With a slow progression of the disease, the pathogenesis of JD makes diagnosis difficult, more so in the subclinical stage of infection before the clinical signs appear (21). The first clinical signs include gradual weight loss despite normal appetite or, sometimes, increased appetite. During this clinical period, there is a decrease in milk production, accompanied by a concomitant weight loss with a sometimes more fluid consistency of manure. Diarrhea may be intermittent at first, with periods of normal manure consistency leading to chronic diarrhea (2). During the prolonged incubation subclinical period of 4–7 years, scarce clinical signs are observed (22). Difficulty in JD diagnosis is further hindered by host genetics (23), herd management, MAP strain, and infectious dose (24).

MAP employs complex mechanisms to control macrophages, which turn into a duel that lasts for years with unpredictable disease progression. The T helper type 1 (Th1)-mediated response that usually effectively controls non-pathogenic intracellular mycobacterial infections fails for MAP infection (25). The pathogenesis of JD is still under investigation because macrophage-MAP cross talk in the subclinical stage of the disease is partially resolved. It is paramount to consider the study of alternative molecular avenues for identifying the product resulting from bacterial MAP infection that might become potential biomarkers of JD and evolve therapeutic tools.

Previous reports indicate that bacteria interfere with mammalian regulatory RNA expression that is not translated to protein (such as long non-coding RNA–lncRNA) to modify immune signaling, autophagy, or apoptosis machinery (26, 27). lncRNAs are now emerging as important regulators of innate and adaptive immune responses (28–30). In humans, while they are widely investigated in aging, cancers, and epigenetics (31–33), there is growing evidence that lncRNAs interfere in the pathogenetic mechanisms of multifactorial disease like Crohn's disease and inflammatory bowel disease (34, 35).

In bovine, few studies have examined the occurrence of lncRNA in tissues, including muscle, skin, various tissues, and the mammary gland (36–40). Previous studies also report that lncRNA is involved with host cell response toward bacterial infections, including paratuberculosis (27, 41). LncRNA is unique from other RNA based on size (>199 nucleotides) and limited evidence of protein-coding potential (36, 37, 42–44). However, lncRNA's novel nature means there is no consensus on the best way to classify the protein-coding and non-coding potential of the lncRNA, so we use both logistic regression (45) and multilayered neural networks (46). The former implements human-designed features, such as open reading frame (ORF) length and integrity, GC content, and hexamer usage bias, whereas the latter identifies multilayered deep patterns solely on sequence information.

This study aims to use available deep learning and logistic regression approaches to study lncRNA associated with MAP in Canadian Holstein and provide novel insight into lncRNA's regulatory function in macrophages of dairy cattle during MAP infection. To this end, we investigated the presence of potentially novel lncRNA candidates and their role in MAP infection using RNA sequencing.

## Materials and Methods

### Cow Selection and Johne's Disease Diagnosis

According to the Canadian Council on Animal Care guidelines for institutional animal use, we carried out all animal procedures and obtained ethical approval for the study from the Agriculture and Agri-Food Canada Animal Ethics Committee (protocol 362). To select cows for RNA-Seq, we analyzed fecal and blood samples from 15 commercial dairy herds positive for JD located in the province of Quebec, Canada, as described in the companion project (47).

Briefly, from each herd, we sampled cows twice yearly. The cows were older than 24 months to be enrolled in the study. They had calved twice or more at culling. We collected one fecal sample of a volume equivalent of 100 mL using a single-use veterinary glove. Consecutively, we also drew two blood samples per cow in dry tubes for serum collection (SST Serum Separation Tubes 8.5 mL; BD Biosciences, Ontario, Canada). Within 1 h of sampling, the tubes were centrifuged at 1,300 × g at 20°C for 10 min and kept at 4°C during the transport to the laboratory. Sera were collected and then stored at −80°C until ELISA analysis. According to the manufacturer's instructions, we processed sera using the IDEXX MAP Ab test kit (IDEXX Laboratories, USA). As described by Collins (48), we transformed optical density values into sample-to-positive (S/P) ratios and selected samples with an S/P ratio of at least 55% as positive. The presence of MAP in feces was tested by qPCR, and JD cows were confirmed infectious using the BD MGIT ParaTB culture medium and the BACTEC 960 detection 960 system described in Fock-Chow-Tho et al. (47). Cows that presented concordant serological and fecal culture or qPCR statuses, either positive or negative, were retained. A cow was designated JD (+) when a fecal culture and ELISA were positive at least two sampling periods. During macrophage analysis, the mean age of JD(–) cows was 6.4 ± 1.5 years, and 5.1 ± 1.8 years for the JD(+) cows. While JD(+) cows were promptly culled, the JD(–) cows were kept on-farm for >7 years to confirm their status definitively. In total, we selected 66 cows for RNA-Seq analysis, of which 33 were JD(+), and 33 were JD(–).

### RNA Isolation, Library Preparation, and Sequencing

The monocyte-derived macrophages (MDM) were prepared in the absence of FBS and granulocyte-macrophage colony-stimulating factor (GM-CSF), or M-CSF, to avoid activation or bias in the differentiation toward M1 or M2 polarization, as described in Ariel et al. (49). Freshly isolated monocytes were confirmed exempt of MAP from both JD(+) and JD(–) cows, confirmed using qPCR and fluorescent microscopy. DNA was extracted from adherent monocytes, MDM, and PBMC using ZR Fecal DNA MiniPrep kit (Zymo Research Corp., Irvine, CA, USA), and qPCR was performed using the VETMAX Gold MAP Detection Kit (Life Technology Inc., Burlington, Ontario, Canada) as described previously (47). The absence of MAP in JD(+) and JD(-) MDM was also confirmed *in vitro* using fluorescence microscopy as described (49). To profile the lncRNA in resting macrophages (CTL, i.e., non-infected) and in response to MAP infection, the MDM were also infected with MAP. Our previous experimental design was used: 1 h, 4 h, 8 h, and 24 h post-infection with MAP at the multiplicity of infection of 10 (49).

In summary, we extracted total RNA from MDM from each experimental time point (CTL and MAP-infected at 1, 4, 8 h, and 24 hpi) in 66 cows [33 JD(–) and 33 JD(+)] using the RNeasy kit (Qiagen) total RNA extraction protocol. We quantified the RNA yield using a NanoDrop spectrophotometer (Thermo Fisher) and assessed RNA quality using the Bioanalyzer RNA 6000 kit (Agilent Technologies). We used the Ribo-Zero Gold kit to remove ribosomal RNA and Illumina TruSeq Stranded Total mRNA Sample Preparation kit (Illumina) to generate cDNA libraries. After quality control (size and absence of primer dimers) and qPCR library quantification [Kappa Library Quantification kit (Roche)], we performed paired-end sequencing using the Illumina HiSeq 2500 platform running HiSeq Control Software (v2.2.68). A subset of the sequencing data from the 66 cows is available from the Gene Expression Omnibus repository (accession number GSE98363). All processes followed manufacturer recommendations.

### Transcriptome Assembly, Novel lncRNA Prediction, and Differential Expression (DE) Analysis

**Figure 1:**
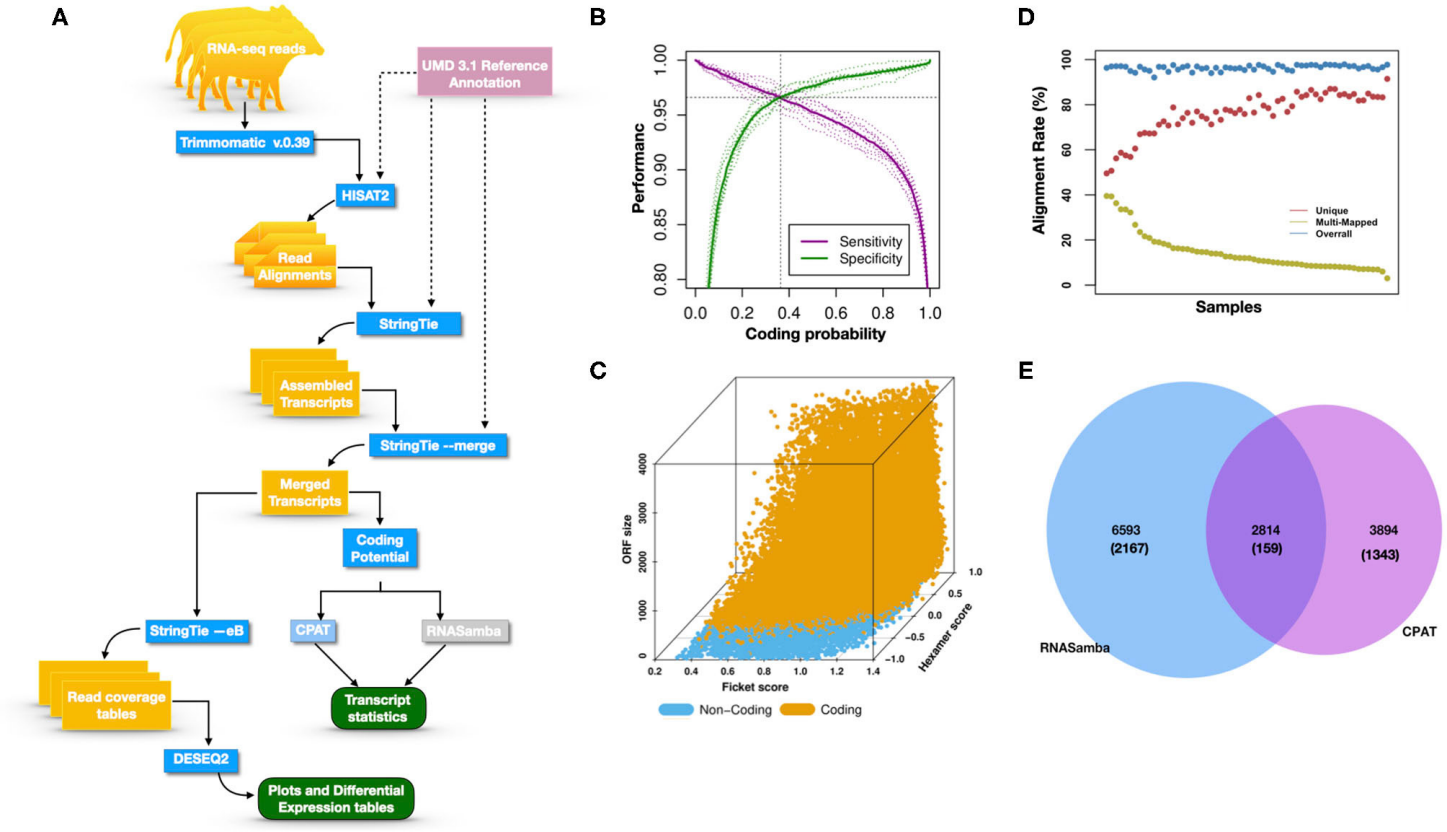
**(A)** The study workflow from raw fastq to lncRNA identification and DE of the nearest genes. **(B)** Two-graph receiver operating characteristic curve (ROC) to determine the optimal coding-probability cutoff value. **(C)** Combinatorial effects of Fickett score, hexamer score, and ORF size on coding transcripts (brown dots) and non-coding genes (blue dots). **(D)** Mapping statistics (vertical axis) showing unique (red), multi-mapped (yellow-green), and overall (blue) alignment rate for all cows (horizontal axis). **(E)** Snapshot of lncRNA statistics identified by two tools with known lncRNAs in parentheses.

Figure 1A illustrates the steps of transcriptome assembly. Illumina adapter sequences were trimmed from each RNA-Seq read using Trimmomatic (V0.39), keeping reads longer than 36 bp and with a Phred score ≥30. Reads were mapped to UMD 3.1.1 bovine genome assembly using HISAT2 (v2.2.0) and transcripts assembled using StringTie (v2.1.0). We merged assembled transcripts from all cows using the—merge option of StringTie, resulting in non-redundant assembled transcripts. Using Gffcompare (v0.10.1), transcripts were then compared with Ensembl bovine gene annotation (release 94) to identify transcripts overlapping with known protein-coding and non-coding regions. To identify lncRNA, we used the transcript classification codes of Gffcompare to select transcripts categorized as “u” and with a length of ≥200 nt.

Transcripts were analyzed using two approaches: (1) estimation of transcript coding probability and (2) differential expression (DE) analysis. Coding probability was estimated using two tools: RNAsamba (46) and CPAT (v2.0.0) (45). Both tools were tested using the *Bos taurus* dataset of known and unknown protein-coding sequences to train the models. RNAsamba computes RNA sequences' coding potential using a neural network classification model resulting in sequences classified as coding or non-coding based on an estimated coding score. The approach of CPAT uses a logistic regression model. Also, CPAT evaluates each base's unequal content frequency and asymmetrical distribution in the positions of codons in one sequence, i.e., the Fickett score and usage bias of adjacent amino acids in proteins, namely, the hexamer score. The models' respective outputs were evaluated using 20-fold cross-validation to determine the coding probability cutoff (Figures 1B,C). Using usearch (50), transcripts with a coding probability of ≤ 0.4 and ORF ≤ 300 bp were selected. Sequences were further filtered out if they blasted against the Swiss-Prot database (e < 1 × 10^−05^). The final dataset was compared to the NONCODEV5 database (51). Those annotated were qualified as known bovine lncRNA, and the remaining isoform transcripts (code = “j”) were classified as novel lncRNA.

We used DEseq2 (v1.26.0) for DE analysis using raw read counts of each sample from the final retained dataset. DESeq2 calculates each sample size factor to correct for library size and RNA composition bias (52). We considered lncRNA as truly expressed if normalized counts ≥5 in at least 10% of our libraries and an FDR < 0.5. To explore the functions of significant DE lncRNA, we used bedtools to obtain the genes 100 kb of each lncRNA. We performed gene ontology (GO) term enrichment analysis using the Panther classification system (53) and Reactome pathways (54). Significance was expressed as *P*-value, with a lower *P*-value indicating higher significance.

## Results and Discussion

In most dairy cows, JD progression is slow, primarily due to the ability of MAP to lodge in intestinal tissue-resident phagocytic cells and escape the immune system's surveillance. Myeloid cells, including monocytes, macrophages, and neutrophils, work in concert with lymphoid cells to initiate and amplify innate and adaptive immunity. Previous reports indicate that lncRNA plays a significant role in regulating the immune response toward several bacterial pathogens known to induce *Mycobacterium tuberculosis* infection in human macrophages (55). We hypothesized that lncRNA could be part of the mechanisms employed by MAP to control macrophages. The current study provides information (1) on potentially lncRNA-targeted genes affected by MAP infection to weaken the host and (2) on the biological pathways that might lead to susceptibility to MAP infection. In our study, RNA-Seq data from macrophages of 33 cows diagnosed JD positive (+), and 33 JD negative (–) cows were used, among which the study of differential gene expression of 12 cows was previously described (49).

### Prediction of lncRNA From Expressed in Bovine Macrophages

To investigate the potential role of lncRNA in macrophages for JD susceptibility, we used RNA-Seq data of macrophages from JD(+) and JD(–) cows. Previous studies have demonstrated the importance of a strong correlation between read alignments and accurate transcript assembly and quantification since misaligned reads usually decrease the number of reconstructed genes (56). In our previous study (49), we analyzed the differentially expressed (DE) genes in macrophages from JD(+) and JD(–) cows using a TopHap-Cufflink pipeline. In the current study, we used HISAT2 because this program, while aligning RNA-Seq reads to the genome, discovers transcript splice sites and provides an accurate representation of all transcript isoforms, creating a rigorous representation of lncRNA (56).

Identifying and inferring lncRNA's biological role is challenging, more so for dairy cattle, where the functional annotation of lncRNA is limiting (57). This study implemented a computational pipeline based on the “new Tuxedo” package (56). As illustrated in Figure 1A, we used HISAT2, StringTie, and Gffcompare to align transcripts to the reference genome, assemble the transcripts, and produce transcript statistics, thus allowing us to obtain results comparable to previous lncRNA studies in cattle (38, 40, 58). Furthermore, we obtained promising alignment statistics (>92% concordant alignment for all samples, Figure 1D) with HISAT2, which was more accurate compared to its preceding software (59, 60), and exhibited a faster search algorithm (61). After mapping a minimum of 13 million paired reads per cow to the UMD 3.1.1 bovine reference genome, on average, 71% were uniquely aligned, 14% were multi-mapped, and the average overall alignment rate was 92% (Figure 1D). Of the lncRNAs identified in this study, 45.05% had an average length of 600 bp long with a range of 200–1,000 bp, most of which were mostly intergenic. This result concurred with previous reports that lncRNAs are mainly located between genes (i.e., intergenic) with a smaller overlap within genic regions (42, 44). Moreover, though previous studies indicate intergenic lncRNA may act in cis or trans to regulate gene activities (62, 63), the ever-continuing curation of the bovine functional lncRNA annotation posed a challenge to study how the identified lncRNA may act in trans to regulate distant genes.

**Figure 2:**
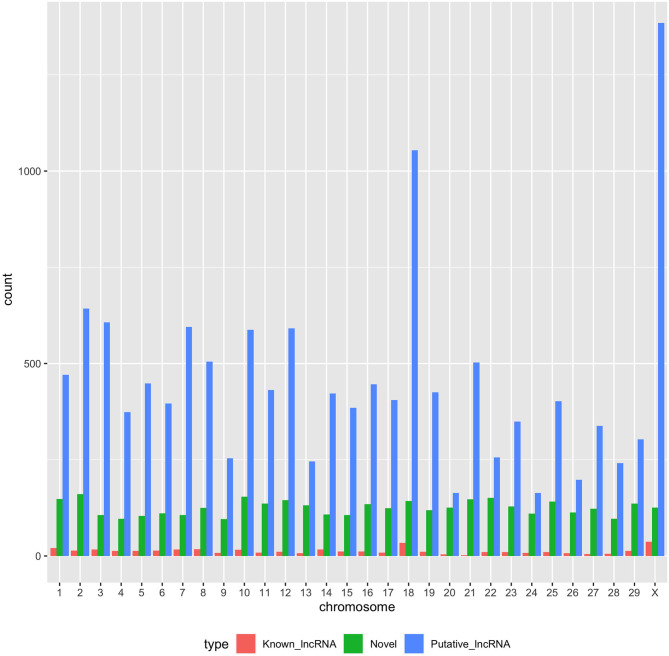
Distribution of putative, known, and novel lncRNAs in chromosomes 1–29 and X.

With the low multi-mapped read rate and high overall alignment rate, we were confident that false-positive alignments would not disrupt StringTie's flow algorithm and skew the expression estimates of assembled transcripts. Parsed mapped files identified 47,683 potential transcripts, of which 13,301 were putative lncRNA (i.e., had a code =“u”). CPAT and RNAsamba predicted 3,894 and 6,593 as non-coding transcripts, respectively, with an intersection of 2,814 non-coding transcripts (Figure 1E). Most of the lncRNA were <1,000 base pairs, with the shortest lncRNA being on chromosomes 12, 26, 27, and X, with *ENSBTAG00000046640, LBX1, ERI1*, and *AMELX* being the closest genes, respectively (Table 1). Chromosomes X and 18 report the highest number of lncRNAs identified in bovine macrophages, with 1,385 and 1,054 lncRNA, respectively (Figure 2).

**Table 1:** Location of the highest DE lncRNAs in macrophages from JD(+) vs. JD(–) cows identified on *Bos taurus* autosomes 1–29 and chromosome X.

**Chrom^**a**^**	**Start**	**End**	**Length (kb)^**b**^**	**Closest gene (kb)^**c**^**	**Gene^**d**^**	**Mean^**e**^**	**Fold change^**f**^**	**Wald statistic^**g**^**	***P*-value^**h**^**
1	128523379	128576527	53.15	—	*ZBTB38*	10198.41	0.25 (±0.08)	3.23	1.25e-03
2	59365700	59366021	0.32	10.25	*HNMT*	43.3	−0.23 (±0.15)	−1.48	1.30e-01
3	120249265	120249575	0.31	20.37	*ANKMY1*	56.44	−0.64 (±0.22)	−2.91	3.65e-03
4	7843837	8440753	596.92	49.92	*FZD1*	3159.91	0.53 (±0.18)	2.92	3.45e-03
5	28846068	28846987	0.92	0.12	*LETMD1*	111.3	−0.34 (±0.14)	−2.36	1.00e-02
6	52972592	52972837	0.24	964.81	*PCDH7*	8.3	1.12 (±0.54)	2.09	3.00e-02
7	21107248	21107521	0.27	7.63	*CREB3L3*	35.87	−0.59 (±0.21)	−2.78	5.51e-03
8	23054554	23054855	0.3	0.01	*ENSBTAG00000039963*	15.44	−1.41 (±0.79)	−1.79	7.00e-02
9	89536157	89567424	31.27	—	*bta-mir-2285e-2*	1033.91	1.23 (±0.36)	3.4	6.65e-04
10	98235095	98343170	108.08	—	*FLRT2*	218.23	1.49 (±0.42)	3.51	4.45e-04
11	43739783	43741807	2.02	24.14	*RF00026*	10148.11	0.18 (±0.1)	1.86	6.00e-02
12	52352608	52352826	0.22	0.28	*ENSBTAG00000046640*	18.24	−0.49 (±0.22)	−2.19	2.00e-02
13	46917583	46917875	0.29	82.89	*LARP4B*	14.21	−0.32 (±0.23)	−1.37	1.70e-01
14	57874677	57874911	0.23	89.6	*TMEM74*	5.22	1.09 (±0.41)	2.66	7.89e-03
15	22944062	22952348	8.29	23.66	***PTS***	5.9	0.67 (±0.29)	2.3	2.00e-02
16	44005434	44008857	3.42	0.77	*DFFA*	2764.31	−0.34 (±0.19)	−1.79	7.00e-02
17	6578427	6636825	58.4	—	*SH3D19*	129.96	0.64 (±0.27)	2.34	1.00e-02
18	65524855	65525608	0.75	1.97	*ENSBTAG00000013020*	314.44	0.36 (±0.11)	3.29	9.86e-04
19	43812878	43813292	0.41	0.64	***NBR1***	1843.89	0.29 (±0.15)	1.94	5.00e-02
20	5251758	5252057	0.3	13.05	***BOD1***	12.56	−1.23 (±0.37)	−3.35	8.22e-04
21	14494659	14522280	27.62	10.08	*ENSBTAG00000048002*	18545.49	0.21 (±0.12)	1.8	7.00e-02
22	58285995	58286273	0.28	0.45	*CCDC174*	9.15	1 (±0.3)	3.39	6.97e-04
23	9006094	9006521	0.43	1.1	*ANKS1A*	547.63	−0.2 (±0.1)	−1.93	5.00e-02
24	42014626	42014991	0.36	6.48	***TWSG1***	8.31	−1.58 (±0.04)	−4.19	2.81e-05
25	32467533	32467886	0.35	—	*RF00002*	25187.9	0.5 (±0.04)	1.23	2.10e-01
26	21892569	21892785	0.22	1.62	*LBX1*	8.22	−0.75 (±0.01)	−1.28	2.00e-01
27	24191077	24191301	0.22	1.82	***DPH1***	27.11	1.11 (±0.3)	3.77	1.63e-04
28	280436	280721	0.28	0.76	*CCSAP*	108.71	0.28 (±0.01)	1.42	1.50e-01
29	30263006	30263284	0.28	44.06	*KIRREL3*	1.65	−1.27 (±0.5)	−2.35	1.00e-02
X	137567452	137567677	0.22	10.99	*AMELX*	2.4	−1.88 (±0.6)	−2.87	4.07e-03

a *Chrom: Bos taurus chromosome*.

b *Length (kb): length of the lncRNA transcript calculated as the difference between the start and end positions expressed as thousand base pairs*.

c *Closest gene (kb): the distance to the closest coding gene*.

d *Gene: the gene closest to the significant transcript*.

e *Mean: the average of normalized counts for all cows*.

f *Fold change: for a particular gene, the log_2_ fold change of −1 for cows scored as JD(+) vs. JD(–) cows means that Mycobacterium avium subspecies paratuberculosis (MAP) infection induces a DE level of 2^−1^ in JD(+) macrophages compared to JD(–) macrophages*.

g *Wald statistic: Results from a likelihood ratio test comparing the estimated standard error of a log_2_ fold change to test if it is equal to zero between the cow status (negative, positive)*.

h *P-value: False discovery rate adjusted P-value at 0.05*.

### Novel lncRNA in Bovine Macrophages

Among the 16,970 lncRNA predicted by the two tools, 3,669 were novel non-coding transcripts, and 385 were known lncRNA. Interestingly, most novel lncRNAs identified in bovine macrophages were mapped on chromosomes X and 18 (Figure 2 and Supplementary File 1). Chromosomes 26 and 28 had the least number of novel non-coding transcripts, with 148 and 161 lncRNAs, respectively (Supplementary File 1). CPAT predicted a smaller number of overall non-coding transcripts, but 35% of these were novel, whereas RNAsamba predicted a larger number of non-coding transcripts, of which 33% were novel lncRNA (Figure 1E).

### Differential Expression (DE) of lncRNA in Macrophages From JD(+) and JD(–) Cows

**Figure 3:**
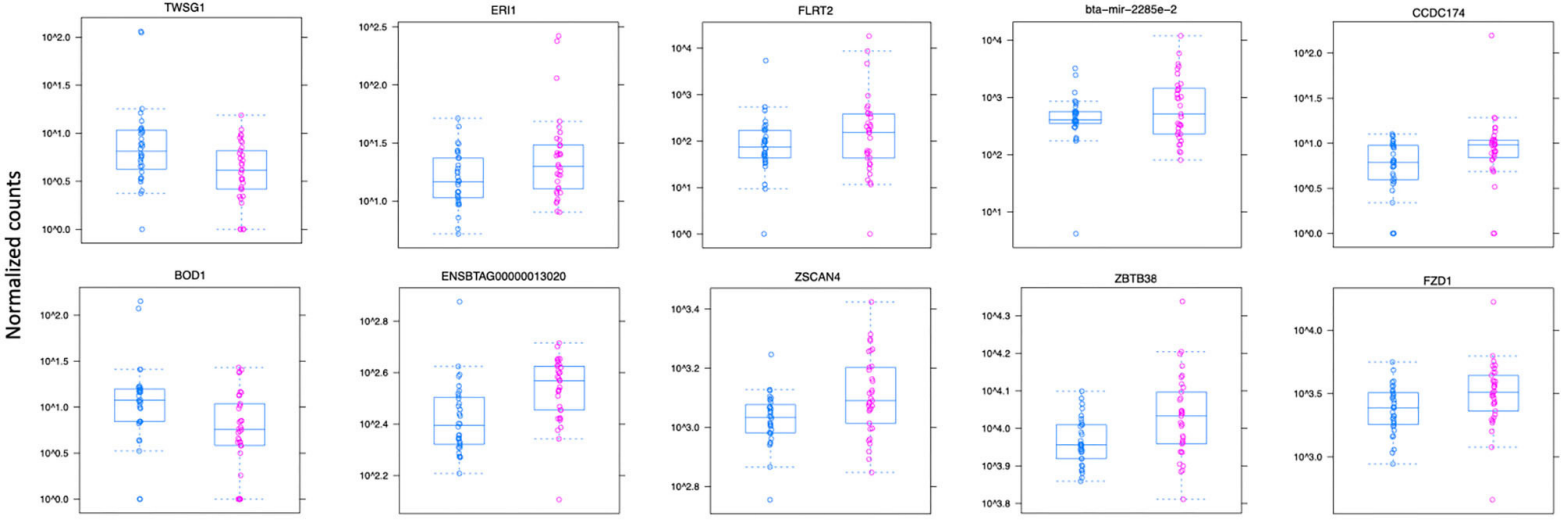
Normalized count distributions in JD(+) macrophages (purple) and JD(–) macrophages (blue) for 10 highly expressed genes close to lncRNA transcripts.

The differential expression (DE) analysis method usually has the most substantial impact on results (64, 65). For this study, we used DESeq2 to study DE of lncRNA detected in macrophages from JD(+) and JD(–) cows. DESeq2 performs robustly in comparison to other existing DE tools (66). As presented in Table 1 and Figure 3, DE analysis using the two predictor tools identified the lncRNA having the greatest significance (*P* < 0.05) for all chromosomes. The two longest DE lncRNAs were on chromosomes 4 and 10, within a 0.5-Mb region (*P* = 3.45 × 10^−03^), at 49 Kb from the *FZD1* gene, and within a 0.1-Mb region (*P* = 4.45 × 10^−04^) close to the *FLRT2* gene, respectively (Table 2 and Figure 3). Interestingly, the Frizzled Class Receptor 1 gene (*FZD1*) was DE in our previous study, where fold change was estimated using FPKM: *FZD1* was 15.56 times more expressed in macrophages from JD(+) cows than JD(–) macrophages (49). The gene encodes a transmembrane domain protein acting as a receptor for Wnt signaling proteins, which are essential for regulating pro-inflammatory cytokines in response to bacteria and mycobacterial infection (67).

**Table 2:** The top 10 most significant DE lncRNAs in macrophages ranked by the FDR corrected *P*-value.

**Chromosome**	**Start**	**End**	**Dispersion^**a**^**	**Length (kb)^**b**^**	**Closest gene (kb)^**c**^**	**Gene^**d**^**	**Mean^***e***^**	**Log2 FC^**f**^**	**Wald statistic^**g**^**	***P*-value^**h**^**
24	42014626	42014991	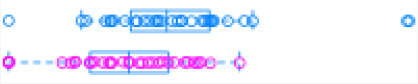	0.36	6.48	***TWSG1***	8.31	−1.58 (± 0.4)	−4.19	2.81e-05
27	24191077	24191301	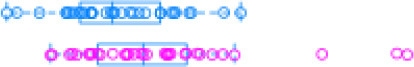	0.22	1.82	***DPH1***	27.11	1.11 (± 0.3)	3.77	1.63e-04
10	98235095	98343170	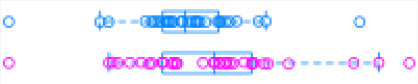	108.08	—	*FLRT2*	218.23	1.49 (± 0.4)	3.51	4.45e-04
9	89536157	89567424	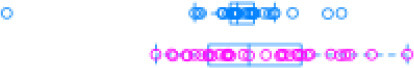	31.27	—	*AKAP12*	1033.91	1.23 (± 0.4)	3.4	6.65e-04
22	58285995	58286273	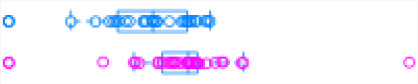	0.28	0.45	*CCDC174*	9.15	1 (± 0.3)	3.39	6.97e-04
20	5251758	5252057	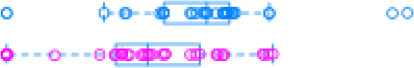	0.3	13.05	***BOD1***	12.56	−1.23 (± 0.4)	−3.35	8.22e-04
18	65524855	65525608	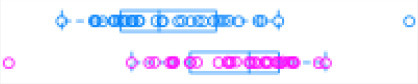	0.75	1.97	*ZNF814*	314.44	0.36 (± 0.1)	3.29	9.86e-04
18	65255175	65265109	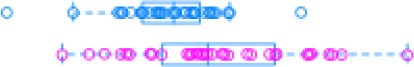	9.93	13.44	*ZSCAN4*	1216.85	0.31 (± 0.1)	3.29	1.01e-03
1	128523379	128576527	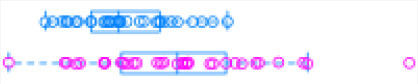	53.15	—	*ZBTB38*	10198.41	0.25 (± 0.1)	3.23	1.25e-03
4	7843837	8440753	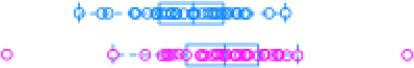	596.92	49.92	***FZD1***	3159.91	0.53 (± 0.2)	2.92	3.45e-03

a *Dispersion: Visual representation of normalized count dispersion of lncRNA in a specific region of a selected chromosome in JD(+) macrophages (purple) and JD(–) macrophages (blue)*.

b *Length (kb): Length of the lncRNA transcript calculated as the difference between the start and end positions expressed as thousand base pairs*.

c *Closest gene (kb): The distance from the significant lncRNA to the closest coding gene*.

d *Gene: The closest gene to the significant lncRNA transcript. Genes expressed in bovine macrophages are in bold characters*.

e *Mean: The average of normalized counts for all cows*.

f *Fold change: For a particular gene, the log_2_ fold change of −1 for cows scored as Positive vs. cows scored as Negative means that infection of Mycobacterium avium subspecies paratuberculosis (MAP) for JD(+) cows induces a multiplicative change in macrophages, observed as the gene expression level of 2^−1^ compared to the macrophages from JD(–) cows*.

g *Wald statistic: Result of a likelihood ratio test comparing the estimated standard error of a log_2_ fold change to test if it is equal to zero between the cow status [JD(–), JD(+)]*.

h *P-value: False discovery rate adjusted P-value at 0.05*.

Ten of the predicted lncRNAs were highly DE at *P* < 0.05 (Table 2). The most highly DE lncRNA was downregulated by ~3-fold change (FC) (Log_2_ −1.58 ±0.4) in JD(+) compared to JD(–) macrophages. This lncRNA is located within a 0.4-Kb region on chromosome 24 and is close to the predicted twisted gastrulation protein homolog one gene (*TWSG1)* (Table 2). *TWSGI* was found expressed in bovine macrophages and, interestingly, was previously significantly downregulated by 0.70 Log_2_FC in response to MAP infection at 4 h post-infection (4 hpi) (49). The second highest DE was an lncRNA of 0.3 Kb located on chromosome 20 with a Log_2_FC of −1.23 (±0.4). The negative Log_2_FC indicates that macrophages from JD(+) cows have 2.35 times fewer lncRNA transcripts than macrophages from JD(–) cows. This lncRNA is 13 Kb away from *Bos taurus* biorientation of chromosomes in cell division 1 gene (*BOD1*). Although *BOD1* is a provisional NCBI gene (accession no. NM_001076200), *BOD1* expression was detected in bovine macrophages (49) but was however not found DE between JD(–/+) groups. Two lncRNAs with the greatest Log_2_FC are located in a 31-Kb region of chromosome 9 (FC = 2.35, *P* = 6.65 × 10^−04^, vicinity of *AKAP12*) and in a 108-Kb region of chromosome 10 (Log_2_FC = 2.81, *P* = 4.45 × 10^−04^, vicinity of *FLRT2*). These lncRNAs' impact on *AKAP12* and *FLRT2* is unlikely because these computationally predicted genes were not found expressed in macrophages (49). However, these lncRNAs' role in JD should not be excluded considering their potential activity on trans-target genes. Overall, there were 255 DE genes within the neighborhood of the lncRNA transcripts (Supplementary Figure 1).

### Functions of lncRNA on the Different Biological Systems in Macrophages

The 255 DE genes were used to explore the functions of significant DE lncRNAs. Part of the Reactome pathways and Panther classification system analysis are presented in Table 3 with some of the Gene Ontology (GO) terms. A detailed report is found in Supplementary File 1. The analysis revealed 14 significant enrichment in the Reactome pathways, among the 1,287 enriched pathways. The highest significant enriched Reactome pathway was the Nuclear Receptor transcription pathway (R-HSA-383280, *P* = 1.69 × 10^−5^). Interestingly, part of this pathway is the Nuclear Receptor Subfamily 3 Group C Member 1 gene (*NR3C1*). *NR3C1* was previously found expressed in bovine macrophages and being upregulated nearly 2-fold in response to MAP infection (Log_2_FC 0.8; *P* < 0.05) (49). This gene encodes a glucocorticoid receptor and chiefly binds small diffusible signaling molecules in the cytoplasm. Upon ligand binding, it migrates to the nucleus to bind glucocorticoid response elements in the promoters of glucocorticoid-responsive genes (68). While its role in JD is not reported, upon glucocorticoid-receptor bindings, various physiological functions, notably those involved in metabolism, inflammatory processes, and stress, are affected (69). This *NR3C1* gene was also identified in the Panther classification system (Table 4 and Supplementary File 1). The *NR3C1* molecular function falls in “glucocorticoid receptor activity” (GO:0004883; *P* = 8.45 × 10^−03^) of the biological activity “glucocorticoid mediated signaling pathway” (GO:0043402) with the highest fold (118.25) enrichment pathway (Table 3). As presented in Table 4, this nuclear receptor *NR3C1* gene was found in “RNA polymerase II cis-regulatory region sequence-specific DNA binding” (GO:0000978; *P* = 1.16 × 10^−04^), in “cis-regulatory region sequence-specific DNA binding” (GO:0000987; *P* = 1.56 × 10^−04^), and in “sequence-specific DNA binding” (GO:0043565; *P* = 1.57 × 10^−04^) pathways (70–72).

**Table 3:** Enriched Reactome pathways using 255 DE lncRNA.

**Reactome pathway name**	**Genes**			**Reactions ratio^**c**^**	**Genes found^**d**^**
	**Found**	**Ratio^**a**^**	***P*-value^**b**^**		
Nuclear receptor transcription pathway	10/107	5.84e-03	1.69e-05	1.53e-04	*ATXN7, **NR3C1***
Deletions in the AMER1 gene destabilize the destruction complex	1/1	6.79e-05	1.66e-02	7.63e-05	*AMER1*
RNA polymerase I promoter escape	4/64	4.35e-03	2.34e-02	1.53e-04	***CBX3**, **H2AFZ**, **POLR1B**, **TWISTNB***
Misspliced GSK3beta mutants stabilize beta-catenin	2/15	1.02e-03	2.66e-02	7.63e-05	*AMER1, **PPP2R5A***
Phosphorylation site mutants of CTNNB1	2/16	1.09e-03	3.00e-02	3.05e-04	*AMER1, **PPP2R5A***
Beta-catenin phosphorylation cascade	2/19	1.29e-03	4.09e-02	3.05e-04	*AMER1, **PPP2R5A***
RUNX3 regulates YAP1-mediated transcription	2/20	6.11e-04	4.48e-02	2.29e-04	*TEAD4, TAZ*
Physiological factors	2/21	8.83e-04	4.89e-02	3.05e-04	*KAT2B, TAZ*
Defective ALG12 causes ALG12-CDG (CDG-1g)	1/3	2.04e-04	4.89e-02	7.63e-05	*ALG12*
MyD88 deficiency (***TLR5***)	1/3	2.04e-04	4.89e-02	7.63e-05	***TLR5***

a *Ratio: the ratio of entities from this pathway to all Reactome entities*.

b *P-value: the result of the statistical test for overrepresentation, for molecules of the type of the results selected which have been corrected for overrepresentation probability*.

c *Reactions ratio: the ratio of reactions from this pathway to all Reactome reactions*.

d *Genes found: some of the genes associated with the Reactome pathway. Genes expressed in bovine macrophages are in bold characters*.

**Table 4:** Gene ontology results using 255 DE lncRNAs.

**GO Id^**a**^**	**Label^**b**^**	**Ratio^**c**^**	***P*-value**	**Fold enrichment^**d**^**	**Genes^**e**^**
**CELLULAR COMPONENT**					
GO:0070062	Extracellular exosome	31.75	3.18e-25	37.53	*ACOT9, ADAD1, **AHR**, AKAP12*, *ALCAM, ALG12, AMELX, AMER1*, *ATXN7, **B4GALT4**, BCL2, BEX3*, ***BOD1**, CBX3, CCDC174, CCNO*, ***CEACAM1**, CMTR2, CNOT11, COQ8B*, *DENND4B, DFFA, **DPH1**, EBF3*, ***FLRT2**, **FOXJ2**, FTH1, **GALNT6**, GLB1L3*, *IRF2BPL, KAT2B, KIRREL3, **KLHL9***, *NR3C1, **NRIP1**, **PLEKHM1**, **POLR1B***, ***SAMD9**, **SLC2A8**, SLC48A1*, ***TWISTNB**, ZSCAN4, **CA8**, UPK1A*
GO:1903561	Extracellular vesicle	27.40	5.63e-24	32.39	
GO:0043230	Extracellular organelle	25.97	1.59e-23	30.71	
GO:0043227	Membrane-bounded organelle	1.36	2.86e-16	1.61	
GO:0043226	Organelle	1.28	6.34e-16	1.51	
GO:0005622	Intracellular	1.21	5.43e-15	1.42	
GO:0031982	Vesicle	2.83	1.57e-13	3.34	
GO:0005737	Cytoplasm	1.32	2.27e-12	1.56	
GO:0043229	Intracellular organelle	1.24	3.84e-12	1.46	
**MOLECULAR FUNCTION**					
GO:0005515	Protein binding	2.29	1.15e-44	2.70	*CCNO, CCR6, CCSAP, **CDA**, CDH11*, *DFFA, DOK5, **DPH1**, EBF3, ENPP2*, *ICE2, IFNAR1, IL12B, IL7R, **IQSEC1***, *MAP3K7CL, **NBR1**, **POLR1B**, **PPP2R5A***, *PRDM1, PRKAR2B, **PTS**, RHOU, RNF122*, *RPE, RRAGB, **SAE1**, SAMD14, SAMD9*, *SBSN, SCGB1A1, SESTD1, SH3D19*, *SLC48A1, SNX24, SNX32, SOD1, SPIN2, SPNS3*, ***STOM**, TLR5, **TMEM19**, TMEM74, TMEM9*, *TOP1, TRMT12, ELMSAN1, **GALNT6**, PCDH7*, *ZNF518A, SAE, **ZNF350**, NBR1, TMEM74*, ***TWSG1**, IQSEC1, **B4GALT4**, CDA, PPP2R5A*
GO:0005488	Binding	1.37	3.18e-24	1.62	
GO:0046872	Metal ion binding	1.57	4.98e-06	1.85	
GO:0043169	Cation binding	1.53	9.96e-06	1.81	
GO:0043167	Ion binding	1.34	1.10e-05	1.58	
GO:0000978	RNA polymerase II cis-regulatory region sequence-specific DNA binding	2.12	1.16e-04	2.5	
GO:0000987	cis-Regulatory region sequence-specific DNA binding	2.07	1.56e-04	2.44	
GO:0043565	Sequence-specific DNA binding	1.81	1.57e-04	2.14	
GO:0003677	DNA binding	1.59	2.03e-04	1.88	
**BIOLOGICAL PROCESSES**					
GO:0043312	Neutrophil degranulation	75.00	7.08e-12	141.90	*ATP6AP2, **CDA**, CEACAM1, FTH1, MMP25*, *STOM, AHR, AKAP12, AMELX, AMER1*, *APPL1, ATXN7, BCL2, BEX3, **BOD1***, *CBX3, CCNO, CNOT11, CREB3L3, CREB3L4*, *DFFA, DOK5, **DPH1**, EBF3, ELMSAN1*, *ENPP2, ERI1, FOXJ2, FZD1, GRSF1*, *H2AFZ, HIVEP3, **HMG20A**, HOMEZ*, ***IBTK**, ICE2, IL12B, **IL7R**, IRF2BPL*, *KAT2B, LARP4B, LBX1, LOXL2, LPXN*, *LSM2, MOSPD1, **MYBL2**, NBR1, NR3C1*, *NRIP1, OXR1, PDE4B, PIK3R4, PKHD1*, *POLR1B, PPP2R5A, PRKAR2B, PUM3*, *RAD50, RAF1, RBBP6, RBM12B, RRAGB*, *SAE1, SCGB1A1, SH3D19, SNX32, SOD1*, *SPIN2, SPTLC2, TAZ, TEAD4, **TIRAP**, **TLR5***, ***PAG1**, SLC2A8, SNRK, ZFAND5, **PRDM1***
GO:0019222	Regulation of metabolic process	1.53	1.25e-10	1.80	
GO:0002283	Neutrophil activation involved in immune response	60.00	4.38e-10	70.95	
GO:0050789	Regulation of biological process	1.22	8.09e-10	1.43	
GO:0031323	Regulation of cellular metabolic process	1.55	9.18e-10	1.83	
GO:0050794	Regulation of cellular process	1.22	2.46e-09	1.44	
GO:0002446	Neutrophil-mediated immunity	42.86	3.20e-09	50.67	
GO:0060255	Regulation of macromolecule metabolic process	1.51	3.81e-09	1.78	
GO:0065007	Biological regulation	1.17	4.04e-09	1.38	
GO:0048518	Positive regulation of biological process	1.53	7.12e-09	1.81	
GO:0051171	Regulation of nitrogen compound metabolic process	1.53	1.04e-08	1.80	
GO:0042119	Neutrophil activation	30.00	2.61e-08	35.47	
GO:0080090	Regulation of primary metabolic process	1.49	3.38e-08	1.76	
GO:0043299	Leukocyte degranulation	28.57	3.47e-08	33.78	

a *Id: gene ontology (GO) symbol accepted by the broader scientific community and sorted by ascending p-value*.

b *Label: GO term accepted by the broader scientific community*.

c *Ratio: the proportion of genes submitted and those found to be associated with the GO term*.

d *Fold enrichment: statistical estimation of obtaining the GO term that is not attributed to random chance*.

e *Genes: a snippet of genes associated with the GO domain, more detailed genes available in Supplementary File 1. Genes found expressed in bovine macrophages are in bold characters*.

A second significant enriched pathway, which includes genes found expressed in bovine macrophages, is the RNA Polymerase I Promoter Escape (Table 3). Interestingly, the *TWISTNB* gene, which encodes the RNA polymerase I subunit F, was upregulated to 2.42 FC (*P* = 5 × 10^−04^) in macrophages from JD(+) cows (49), suggesting increased recruitment of Pol I to rDNA promoters in macrophages from JD(+) cows.

The complete list of GO terms from categories of the Panther classification system, notably molecular function, cellular components, and biological processes, is presented in Supplementary File 1. The biological process category reported 3,144 pathways, of which 2,117 had 2-fold enrichment or better; molecular function reported 148 pathways with 132 having at least a 2-fold enrichment; and 153 pathways had a cellular component with 26 pathways having a 2-fold enrichment or better (Supplementary File 1).

It is well-known for *M. tuberculosis* (73) and suggested for MAP (12) that preventing acidification or fusion of the phagosome to the lysosome is a surviving strategy. Interestingly, several lncRNAs were identified in cellular components associated with lytic vacuole membrane (GO:0098852), lysosomal membrane (GO:0005765), and endocytic vesicle membrane (GO:0030666). Two genes located in the lysosomal membrane were previously found downregulated in macrophages in response to MAP infection, namely, the Solute Carrier Family 2 Member 8 gene (*SLC2A8*) and the Solute Carrier Family 48 Member 1 (*SLC48A1*) (data shown). It is of particular interest that *SLC48A1* encodes to a heme transporter in the context that MAP (70) as for other mycobacteria (71, 72) relies on the host for the acquisition of iron (Fe) which is critical for their growth. Other cellular components were the extracellular exosome (GO:0070062, *P* = 3.18 × 10^−25^) and vesicle (GO:1903561, *P* = 5.63 × 10^−24^) which were the most significant cellular components with a similar high fold enrichment (~30). Interestingly, the histamine N-methyltransferase gene (*HNMT*) was significantly downregulated by 2.7-fold in macrophages infected by MAP at 4 hpi (49).

One of the longest significant lncRNAs, as mentioned above, is in the vicinity of the *FZD1*-encoded transmembrane protein, identified associated with the cellular component focal adhesion (GO:0005925). The molecule *FZD1* functions as protein binding (GO:0005515), a critical Wnt/β-catenin negative feedback loop for repression of Toll-like receptor (TLR)-triggered inflammatory responses (74). This cell receptor in macrophages performs Wnt signaling to regulate pro-inflammatory cytokines in response to bacteria and mycobacterial infection (67). Interestingly, *FZD1* and its ligand Wnt3a are involved in reprogramming *Mycobacterium tuberculosis*-infected macrophages (75). This is particularly interesting for JD while supporting our hypothesis developed based on our previous study (49) and other studies (76) that JD(+) macrophages may be responsive because of tolerance, i.e., epigenetic reprogramming. *FZD1* was found more expressed in macrophages from JD(+) cows than JD(–) macrophages and might explain the phenotypes observed for JD(+) macrophages (49).

### lncRNA's Putative Role of JD-Associated lncRNA in the Immune Response

Though not among the most significant GO terms, immunoreceptor activity (GO:0140375) had a 5.18-fold enrichment. This high fold enrichment is of interest because this function is responsible for receiving a signal and transmitting it in a cell to initiate an immune response during an invasion by a pathogen. The top 10 GO terms involved with biological processes were associated with neutrophils, for instance, neutrophil degranulation (GO:0043312, *P* = 7.08 × 10^−12^), which is involved in regulated exocytosis of secretory granules, neutrophil-mediated immunity (GO:0002446, *P* = 3.2 × 10^−09^), and neutrophil activation (GO:0042119, *P* = 2.61 × 10^−08^). For Mtb infection, secreted products from neutrophils regulate the macrophage activity (77). Since neutrophils are part of the first line of innate immunity of healthy cows (78) and are the second type of cell migrating in lesions of experimentally MAP-infected calves (79), the involvement of these pathways to support neutrophils in their role for pathogen clearance is highly relevant. Interestingly, the “negative regulation of T cell-mediated cytotoxicity” (GO: GO:0001915, *P* = 3.1 × 10^−04^) was among the top enriched biological processes. Both genes associated with this pathway, notably *CEACAM1* and interleukin (IL) 7 receptor gene (*IL7R*), were expressed in bovine macrophages, and most interestingly is IL7R that was up to 7-fold increased in macrophages in response to MAP infection at 4–8 hpi (49). Aberrant plasma IL7 and soluble IL7 receptor levels indicate impaired T-cell response in human tuberculosis (80), and gene expression of IL7R had significant discriminatory power between tuberculosis-positive and -negative patients (81). The relevance of studying the lncRNA associated with IL7R in the pathogenesis of MAP also merits that IL7R is an immune biomarker validated for detecting clinical tuberculosis (82).

Gene ontology (GO) indicated that lncRNA influences, among others, the inflammation, the evidence of which is well-documented for macrophages (83–85). The myeloid differentiation primary response (MyD88) pathway was confirmed to be affected by MAP infection in our previous study (49). Interestingly, previous studies have shown that mice with knocked-out Myd88 are highly susceptible to infection by M. tuberculosis (86), implying that the MyD88-dependent toll-like receptor signaling pathway could be a mycobacterial target for pathogen evasion of host responses. As expected, lncRNA target genes were significantly enriched for biological process GO terms involved in this immune regulation (GO:0034146, GO:0002755). Previous studies report that lncRNA may bind to their target genes; hence, it is unsurprising that the nucleic acid regulation GO terms were enriched (87). GO terms (e.g., GO:0043312, GO:0002446, and GO:0042119) were characterized by pathways associated with neutrophils reported to provide the first line of cellular defense against bacterial colonization in cattle (88).

We observed little overlap between predicted lncRNA candidates and the previously published cattle non-coding RNA. This little overlap may partially be because the lncRNA library is not fully curated in dairy cattle or that previously published non-coding RNA was identified in different tissues since lncRNAs show tissue- and cell-specific expressions. This study does have limitations; one is the incomplete bovine annotation where many unannotated genes exist, both protein- and non-protein-coding. In the current state, this would render the unknown transcripts in our data moot. Collaborative projects may eventually lead to creating a comprehensive map of functional elements in cattle's genome, allowing better identification of potential bovine lncRNA.

A good example is the Functional Annotation of Animal Genomes (FAANG) (89). Previous studies have identified lncRNA using *in vitro* infected macrophages (41). However, our study's novelty aimed to identify genome-wide lncRNAs using primary monocyte-derived macrophages for naturally MAP infected cows.

Our study's rigor also comes from the culture protocol of macrophages that do not bias macrophages' polarization. In Gupta et al. (41), macrophages were cultured in the presence of fetal bovine serum (FBS) and were differentiated in the presence of macrophage colony-stimulating factor (M-CSF). The effect of a culture medium on polarization when supporting monocytes' differentiation has been rigorously documented (90). It turns out that the presence of fetal bovine serum (FBS) and stimulating factors, such as GM-CSF or M-CSF allow a differentiation of the monocytes that activate and polarize them (90). The presence of FBS affects the functionality of monocyte-derived macrophages (91). FBS contains a considerable amount of immunoregulatory cytokines and bioactive molecules, notably the transforming growth factor-β (TGF-β) in notable concentrations (92) that yields a dominant immunosuppressive phenotype in the presence of M-CSF (93). This is all the more important since polarization impacts the lncRNA profile in macrophages (30). An additional novelty of our study is that macrophages were differentiated in the absence of FBS and that no stimulating factors were used to avoid polarization bias. It might explain that we identified 3,669 novel lncRNAs in bovine macrophages compared to 397 novel lncRNAs in the previous study (41).

Dairy and beef studies continuously use underlying biological information of point mutations (e.g., SNP) and genomic features to discover which genomic variants are theoretically enriched (94). The bovine lncRNAs reported here could further extend the underlying biological information by including a new class for genomic variants exclusive in lncRNA regions to enrich bovine health traits.

Although human data studies show that single-exon lncRNAs are more likely to be conserved (95), we did not discriminate against lncRNAs from abundant lowly expressed single-exonic fragments. Furthermore, following stringent filtering criteria based on other genomic features like ORF length, protein-coding potential, and expression levels, we identified 16,970 lncRNAs, 3,669 of which were novel lncRNAs with 255 potential cis target genes.

## Conclusion

In summary, we have provided the lncRNA expression profile of macrophages from JD cows, together with many potential co-regulated candidate protein-coding genes. We have identified 13,589 lncRNAs, 3,669 of which are novel. Among those lncRNAs significantly upregulated in JD(+) macrophages, three were linked (<1 kb) to genes expressed in bovine macrophages, notably *TWSG1, DPH1*, and *BOD1*. Bacteria interfere with mammalian regulatory RNA expression, including lncRNA, to modify molecular and cellular signaling. We identified lncRNAs as having the potential to play a significant role in regulating several cellular activities, including the immune response. Although the mechanisms of MAP are currently unknown, we speculate that many of the identified lncRNAs are essential participants in the bovine innate immune response. At the same time, some could support MAP in macrophages in its duel to escape the surveillance of the immune system that lasts for years.

## Data Availability Statement

The datasets presented in this study can be found in online repositories. The names of the repository/repositories and accession number(s) can be found in the article/Supplementary Material.

## Ethics Statement

The animal study was reviewed and approved by Agriculture and Agri-Food Canada Animal Ethics Committee. Written informed consent was obtained from the owners for the participation of their animals in this study.

## Author Contributions

NB: project administration, supervision, and funding acquisition. AM, OA, and NB: methodology. AM: data curation and formal analysis. AM and NB: writing. All authors provided the input in interpreting the results and have read and agreed to the published version of the manuscript.

## Conflict of Interest

The authors declare that the research was conducted in the absence of any commercial or financial relationships that could be construed as a potential conflict of interest.

## Supplementary Material

Supplementary File 1 **(A)** Distribution of lncRNA per chromosome; **(B)** General Transfer Format (gtf) of identified lncRNA; **(C)** Reactome pathways; **(D)** General biological programs accomplished by the multiple molecular activities (Biological processes; GO terms); **(E)** Molecular-level activities performed by gene products (molecular functions; GO terms), and **(F)** The locations relative to cellular structures in which a gene product performs a function (Cellular component; GO terms).

Supplementary Figure 1 Illustration of gene network including genes within 100 kb of highly expressed lncRNA.
