# Supplementary figures and images for: Identification of Long Non-coding RNA Isolated From Naturally Infected Macrophages and Associated With Bovine Johne's Disease in Canadian Holstein Using a Combination of Neural Networks and Logistic Regression
Source: Front Vet Sci. 2021 Apr 22;8:639053. doi: 10.3389/fvets.2021.639053 (PMC8100051; doi:10.3389/fvets.2021.639053)

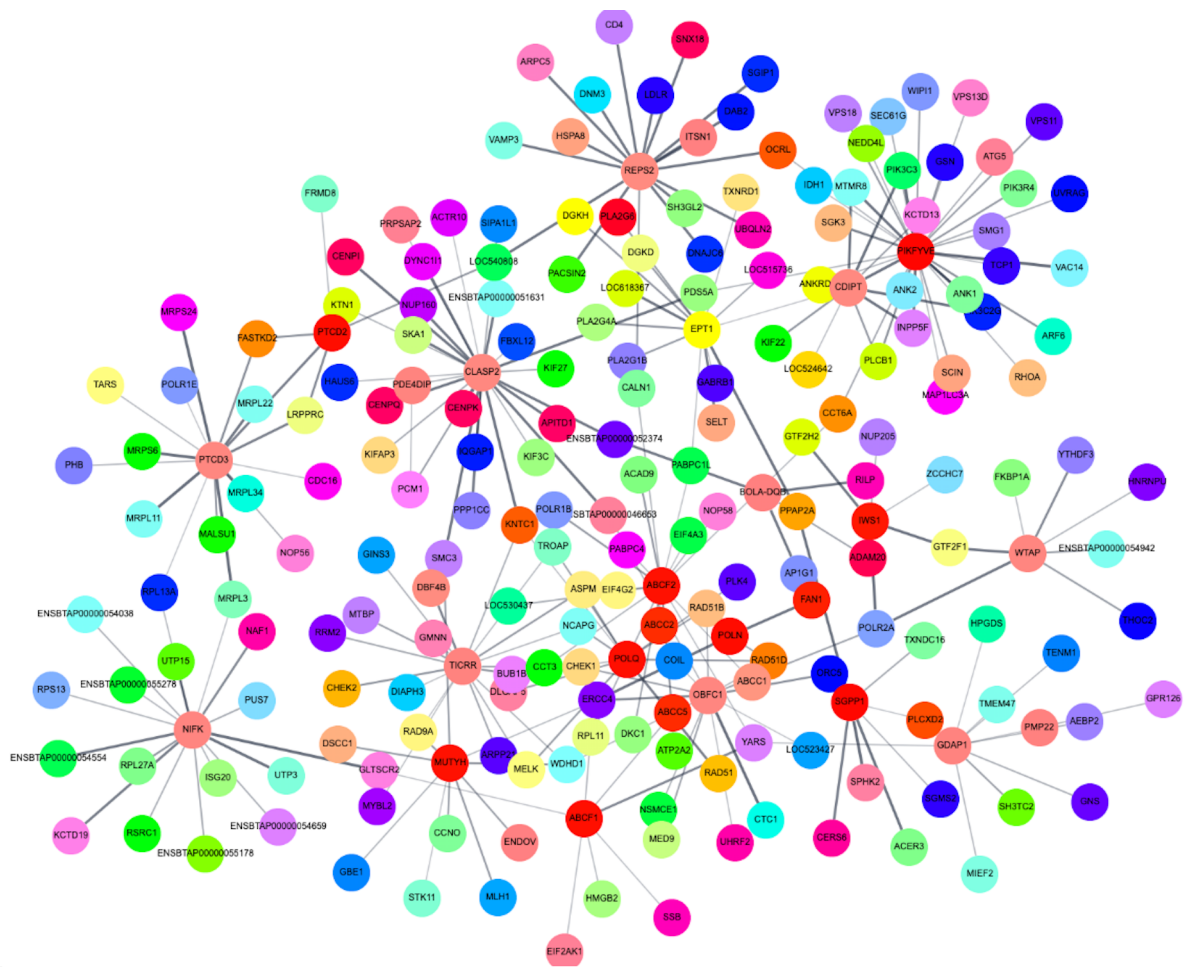

Supplement: Supplementary Figure 1 — Illustration of gene network including genes within 100 kb of highly expressed lncRNA. [file Image_1.pdf]
